# Dermatan sulfate in tunicate phylogeny: Order-specific sulfation pattern and the effect of [→4IdoA(2-Sulfate)β-1→3GalNAc(4-Sulfate)β-1→] motifs in dermatan sulfate on heparin cofactor II activity

**DOI:** 10.1186/1471-2091-12-29

**Published:** 2011-05-29

**Authors:** Eliene O Kozlowski, Paula C Lima, Cristina P Vicente, Tito Lotufo, Xingfeng Bao, Kazuyuki Sugahara, Mauro SG Pavão

**Affiliations:** 1 Laboratório de Bioquímica e Biologia Celular de Glicoconjugados, Hospital Universitário Clementino Fraga Filho and Programa de Glicobiologia, Instituto de Bioquímica Médica, Universidade Federal do Rio de Janeiro, Rio de Janeiro, RJ 21941-913, Brasil; 2 Department of Anatomy, Cellular Biology, Physiology and Biophysics, Instituto de Biologia, Universidade Estadual de Campinas, São Paulo, Brasil; 3 Laboratório de Ecologia Animal, Instituto de Ciências do Mar, Universidade Federal do Ceará, Av. Abolição 3207, Fortaleza, CE, 60165-081, Brasil; 4 Department of Biochemistry, Kobe Pharmaceutical University, Kobe 658-8558, Japan; 5 Glycobiology Unit, Tumor Microenvironment Program, Cancer Research Center, Sanford-Burnham Institute for Medical Research, 10901 North Torrey Pines Road, La Jolla, California 92037, USA; 6 Laboratory of Proteoglycan Signaling and Therapeutics, Hokkaido University Graduate School of Life Science, Frontier Research Center for Post-Genomic Science and Technology Graduate, Sapporo 001-0021, Japan

## Abstract

**Background:**

Previously, we have reported the presence of highly sulfated dermatans in solitary ascidians from the orders Phlebobranchia (*Phallusia nigra*) and Stolidobranchia (*Halocynthia pyriformis *and *Styela plicata*). Despite the identical disaccharide backbone, consisting of [→4IdoA(2S)β-1→3GalNAcβ-1→], those polymers differ in the position of sulfation on the N-Acetyl galactosamine, which can occur at carbon 4 or 6. We have shown that position rather than degree of sulfation is important for heparin cofactor II activity. As a consequence, 2,4- and 2,6-sulfated dermatans have high and low heparin cofactor II activities, respectively. In the present study we extended the disaccharide analysis of ascidian dermatan sulfates to additional species of the orders Stolidobranchia (*Herdmania pallida*, *Halocynthia roretzi*) and Phlebobranchia (*Ciona intestinalis*), aiming to investigate how sulfation evolved within Tunicata. In addition, we analysed how heparin cofactor II activity responds to dermatan sulfates containing different proportions of 2,6- or 2,4-disulfated units.

**Results:**

Disaccharide analyses indicated a high content of disulfated disaccharide units in the dermatan sulfates from both orders. However, the degree of sulfation decreased from Stolidobranchia to Phlebobranchia. While 76% of the disaccharide units in dermatan sulfates from stolidobranch ascidians are disulfated, 53% of disulfated disaccharides are found in dermatan sulfates from phlebobranch ascidians. Besides this notable difference in the sulfation degree, dermatan sulfates from phlebobranch ascidians contain mainly 2,6-sulfated disaccharides whereas dermatan sulfate from the stolidobranch ascidians contain mostly 2,4-sulfated disaccharides, suggesting that the biosynthesis of dermatan sulfates might be differently regulated during tunicates evolution. Changes in the position of sulfation on N-acetylgalactosamine in the disaccharide [→4IdoA(2-Sulfate)β-1→3GalNAcβ-1→] modulate heparin cofactor II activity of dermatan sulfate polymers. Thus, high and low heparin cofactor II stimulating activity is observed in 2,4-sulfated dermatan sulfates and 2,6-sulfated dermatan sulfates, respectively, confirming the clear correlation between the anticoagulant activities of dermatan sulfates and the presence of 2,4-sulfated units.

**Conclusions:**

Our results indicate that in ascidian dermatan sulfates the position of sulfation on the GalNAc in the disaccharide [→4IdoA(2S)β-1→3GalNAcβ-1→] is directly related to the taxon and that the 6-O sulfation is a novelty apparently restricted to the Phlebobranchia. We also show that the increased content of [→4IdoA(2S)β-1→3GalNAc(4S)β-1→] disaccharide units in dermatan sulfates from Stolidobranchia accounts for the increased heparin cofactor II stimulating activity.

## Background

Dermatan sulfate (DS) is a heterogeneous glycosaminoglycan (GAG), formed by repeating disaccharide units consisting of 4-linked glucuronic acid, 1,3-linked N-acetyl galactosamine ([→4GlcAβ-1→3GalNAcβ-1→]). The extensive heterogeneity of the polymer results from variation in the degree of epimerization on carbon 5 of glucuronic acid, O-sulfation on carbon 4 or 6 of N-Acetyl galactosamine (GalNAc) and on carbon 2 of iduronic acid (IdoA) [1]. It has been shown that regions enriched in [→4IdoA(2S)β-1→3GalNAc(4S)β-1→] units in DS bind to heparin cofactor II (HCII) (2S and 4S represent 2-O- and 4-O-sulfate, respectively), enhancing the HCII-induced inhibition of thrombin [2,3].

Previously, we reported the presence of highly sulfated DSs in solitary ascidians from orders Phlebobranchia (*Phallusia nigra*) and Stolidobranchia (*Halocynthia pyriformis *and *Styela plicata*) [4-6]. These polymers are composed by the same disaccharide backbone, consisting of [→4IdoA(2S)β-1→3GalNAcβ-1→], but differ in the position of sulfation on GalNAc, which can be sulfated at carbon 4 or 6 [7]. The DS from the stolidobranchs *H.pyriformis *and *S.plicata *are sulfated at carbon 2 of IdoA and carbon 4 of GalNAc [6] and are potent activators of HCII. On the other hand, the DS from the phlebobranch *P.nigra *is sulfated at carbon 2 of IdoAc and at carbon 6 of GalNAc, and is a poor activator of HCII [5].

Ascidians (sea-squirts) are an ancient life form with fossils dating back to the Early Cambrian [8,9]. They belong to the phylum Chordata, subphylum Tunicata (Urochordata). Because of their basal position on the chordate phylogeny, ascidians are key to understanding chordate evolution and the origin of vertebrates [10-13]. The traditional classification of the Ascidiacea includes three orders: Aplousobranchia, Stolidobranchia and Phlebobranchia. This traditional view has been disputed by many recent reconstructions of their evolution, gathering evidence of the paraphyly of Ascidiacea, although confirming the monophyly of Tunicata [14]. While all ascidian orders include colonial forms, solitary species are only present in Stolidobranchia and Phlebobranchia [15].

The study of ascidians has demonstrated several ways in which these organisms can inform us about our own development and evolution. To increase our understanding about how DS sulfation evolved in chordates, we analyzed the disaccharide composition of DSs obtained from two species of stolidobranch (*Herdmania pallida *and *Halocynthia roretzi*), and one species of phlebobranch ascidians (*Ciona intestinalis*). The results were further compared with our previous data on DSs from other ascidians of the same orders. In addition, we investigated the ability of the ascidian DSs to potentiate HCII. Our results indicate that in ascidians the position of sulfation on the GalNAc in the disaccharide [→4IdoA(2S)β-1→3GalNAcβ-1→] is directly related to the taxon and that the 6-O sulfation is a novelty apparently restricted to the Phlebobranchia. We also show that the presence of [→4IdoA(2S)β-1→3GalNAc(4S)β-1→] disaccharide units in DSs from stolidobranch ascidians accounts for their increased HCII-stimulating activity.

## Results

### Analysis of the GAGs from *H*. *pallida *and *C. intestinalis*

**Figure 1:**
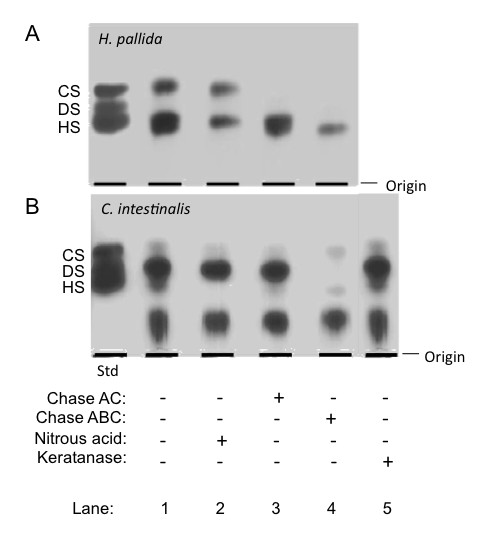
**Isolation of total polysaccharides from *H. pallida *(A) and *C. intestinalis *(B)**. A and B, the polysaccharides extracted from ascidians (~15 mg) before (-) and after (+) incubation with chondroitin AC or ABC lyases and treatment with nitrous acid, were applied to a 0.5% agarose gel and run for 1 h at 100 V in 0.05 M 1,3-diaminopropane/acetate (pH 9.0). The polysaccharides in the gel were fixed with 0.1% N-cetyl-N,N,N-trimethylammonium bromide solution. After 12 h, the gel was dried and stained with 0.1% toluidine blue in acetic acid/ethanol/water (0.1:5:5, v/v). As a standard, a mixture of mammalian glycosaminoglycans containing 10 mg of each chondroitin 4-sulfate (CS), dermatan sulfate (DS) and heparin (Hep) was applied in agarose gel.

A qualitative analysis of the sulfated GAGs extracted from *Herdmania pallida *and *Ciona intestinalis *was carried out by agarose gel electrophoresis, before or after degradation of the glycans with specific GAG-lyases or deaminative cleavage with nitrous acid. Several metachromatic bands with different electrophoretic motilities were observed in the gel of the total GAGs extracted from each ascidian species (Figure 1A and 1B, line 1). With the exception of the very low mobility metachromatic band present exclusively in the gel of *C. intestinalis*, which resists all GAG-degrading enzymes and nitrous acid treatment (Figure 1B), all other bands in the gel of *H. pallida *and *C. intestinalis *disappear after incubation with a specific GAG-degrading enzyme or deaminative cleavage with nitrous acid. Thus, the metachromatic band migrating slightly under that of standard heparan sulfate (HS) in the gels of both *H. pallida *and *C. intestinalis *(Figure 1A and 1B, line 2) disappears after nitrous acid treatment. The band migrating as standard chondroitin sulfate (CS), which is stronger in the gel of *H. pallida*, disappears after incubation with chondroitinase AC (Figure 1A, line 3). In line 4, chondroitinase ABC treatment degrades the top part of the band migrating as HS in figure 1A, and the band migrating at the same position as standard dermatan sulfate (DS) in figure 1B. Overall, these results point to the presence of CS, DS and HS in the ascidians *H. pallida *and *C. intestinalis*, occurring at different proportions, as differences in band densities indicate. In addition, an unidentified glycan, corresponding to the very low mobility band in Figure 1B is also present in *C. intestinalis*.

### Fractionation of *H. pallida *and *C. intestinalis *GAGs

**Figure 2:**
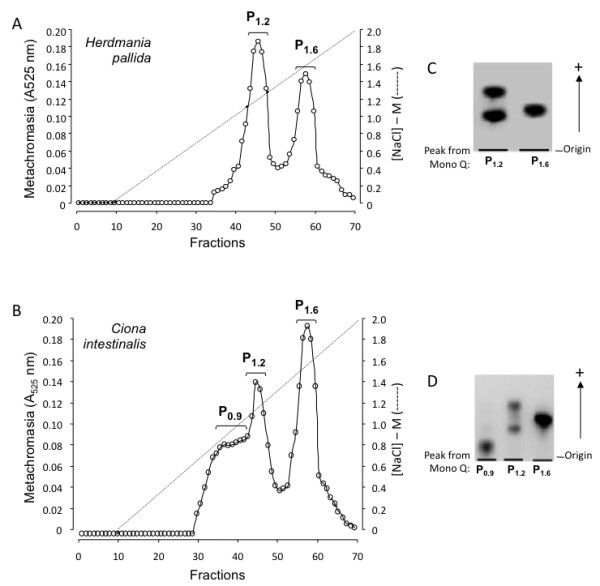
**Purification of the dermatan sulfate (DS) from *H. pallida *(A and B) and *C. intestinalis *(C and D) on a Mono Q-FPLC column**. A and C, the total polysaccharides extracted from ascidians were applied to a Mono Q-FPLC column and purified as described under "Materials and methods". Fractions were assayed by metachromasia (•), and NaCl concentration (- - -). The fractions indicated by horizontal bars were pooled in peaks, denominated P1 and P2 (A) or P1, P2 and P3 (C), dialyzed against distilled water and lyophilized. The vertical arrows indicate the elution of standard mammalian dermatan sulfate (DS) and heparin on Mono Q-FPLC column. B and D, ~15 mg of each peak from Mono Q-FPLC column were applied to a 0.5% agarose gel and run for 1 h at 100 V in 0.05 M 1,3-diaminopropane/acetate (pH 9.0). The polysaccharides in the gel were fixed with 0.1% N-cetyl-N,N,N-trimethylammonium bromide solution. After 12 h, the gel was dried and stained with 0.1% toluidine blue in acetic acid/ethanol/water (0.1:5:5, v/v). As a standard, a mixture of mammalian glycosaminoglycans containing 10 mg each of chondroitin 4-sulfate (CS), dermatan sulfate (DS) and heparin (Hep) was applied in agarose gel.

Total GAGs from *H. pallida *and *C. intestinalis *were applied to an ion-exchange column and eluted with increasing NaCl concentration (Figure 2). Two main metachromatic peaks eluted from the column with 1.2 M and 1.6 M NaCl and were named **P**_**1.2 **_and **P**_**1.6**_, respectively (Figure 2A and 2B). A significant metachromatic shoulder, eluting with 0.9 M NaCl at the 1.2 M-peak of the total GAGs from *C. intestinalis *is also observed (Figure 2B). Qualitative analysis by agarose gel electrophoresis of the peaks eluted from the column before or after (not shown) enzymatic or nitrous acid treatment indicated that **P**_**1.2 **_contains mainly HS (low-mobility band) and CS (high-motility band), whereas **P**_**1.6 **_is composed exclusively of DS. **P**_**0.9 **_is probably the non-GAG sulfated polysaccharide mentioned earlier, since it resists incubation with chondroitinase AC and ABC, keratanase and deaminative cleavage with nitrous acid.

### Characterization of the purified DSs from the ascidians by agarose and polyacrylamide gel electrophoresis

**Figure 3:**
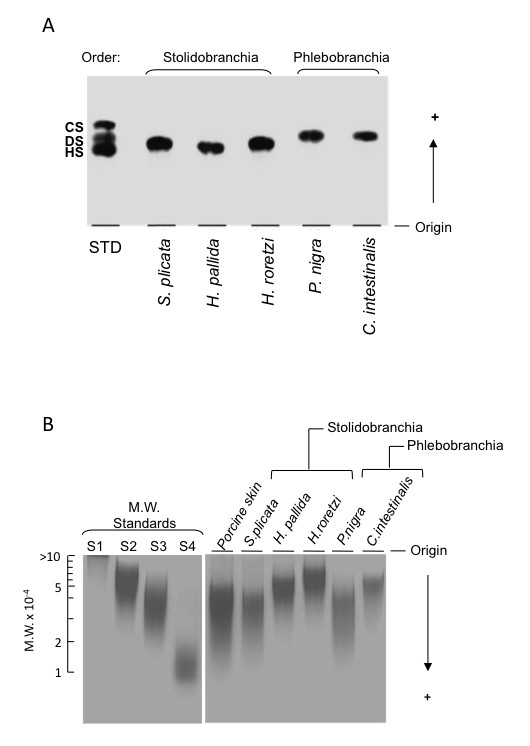
**Agarose and polyacrylamide gel electrophoresis of ascidians and mammalian dermatan sulfate**. A, purified dermatan sulfate (~15 mg) from *S. plicata*, *H. pallida*, *P. nigra *or *C. intestinalis *were applied to a 0.5% agarose gel and run for 1 h at 100 V in 0.05 M 1,3-diaminopropane/acetate (pH 9.0). Glycosaminoglycans were fixed with 0.1% N-cetyl-N,N,N-trimethylammonium bromide solution. After 12 h, the gel was dried and stained with 0.1% toluidine blue in acetic acid/ethanol/water (0.1:5:5, v/v). To a standard, a mixture of mammalian glycosaminoglycans containing 10 mg each of chondroitin 4-sulfate (CS), dermatan sulfate (DS) and heparin (Hep) were applied in agarose gel. B, purified dermatan sulfate (~15 mg) from *S. plicata*, *H. pallida*, *P. nigra *or *C. intestinalis *were applied to 6% 1-mm-thick polyacrylamide gel slab in 0.02 M sodium barbital (pH 8.6) and run for 30 min at 100 V. After electrophoresis the dermatan sulfates were stained with 0.1% toluidine blue in 1% acetic acid and then washed for about 4 h in 1% acetic acid. The molecular mass (M.W.) markers were high molecular mass dextran sulfate (S1, ~500 kDa), chondroitin 6-sulfate (S2, ~54 kDa), chondroitin 4-sulfate (S3, ~36 kDa) and low molecular mass dextran sulfate (S4, ~8 kDa).

The purified ascidian DSs were analyzed by two different electrophoretic systems: agarose gel in diaminopropane buffer, where the electrophoretic motilities of the GAGs is related to their structural characteristic; and polyacrylamide gel in barbital buffer, where the molecules are separated according to their molecular weight. The DSs from *H. pallida *and *C. intestinalis *were analyzed along with that from *Halocynthia roretzi*, which was purified as described in the Methods section, and those from *S. plicata *and *P. nigra*, that were purified as described [5,6]. As shown in figure 3A, the electrophoretic motilities of the DSs from the stolidobranchs *S. plicata*, *H. pallida *and *H. roretzi *were very similar, migrating between DS and HS standards. These motilities differ from those of the DSs from the phlebobranchs *P. nigra *and *C. intestinalis *that migrate higher in the gel, similar to that of DS standard.

Analysis of the ascidian DS by polyacrylamide gel electrophoresis in barbital buffer, revealed that there is not a similar electrophoretic mobility pattern among the DS from different species of the same ascidian order (Figure 3B). The DSs from species of the orders Stolidobranchia and Phlebobranchia differ in molecular weight as well as in polydispersity. The DSs from *S. plicata *and *P. nigra *have the lowest average molecular weight (~30 KDa). On the other hand, the DS from *H. roretzi *has the highest average molecular weight (~50 KDa), whereas the DSs from the *H. pallida *and *C. intestinalis *have an intermediate average molecular weight (~45 KDa). The DS from *S. plicata *and *P. nigra *have high molecular weight and are very polydisperse, similar to what is observed for porcine skin DS. It is interesting to notice that differences in molecular weight do not influence the mobility of the DSs in the agarose gel electrophoresis.

### Disaccharide composition of the ascidian DSs

The purified DSs from *H. pallida*, *H. roretzi *and *C. intestinalis *were exhaustively degraded with chondroitinase ABC. The disaccharides formed (>95%) were separated by gel filtration chromatography, as described in material and methods and analyzed on a strong anion-exchange HPLC.

**Figure 4:**
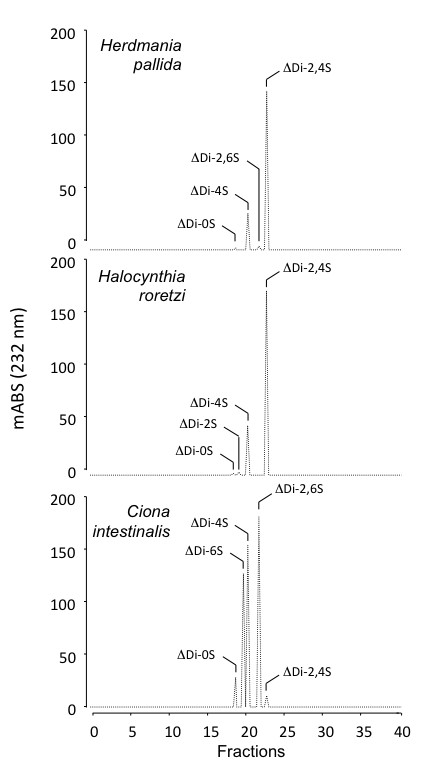
**Strong anion-exchange HPLC analysis of the disaccharides formed by chondroitin ABC lyase digestion of ascidians dermatan sulfate**. A mixture of standards disaccharide (A) and disaccharides formed by exhaustive action of chondroitin ABC lyase on dermatan sulfate from *Herdmania pallida *(B) and *C. intestinalis *(C) were applied to a 25-cm × 4.6-mm Spherisorb-SAX column, linked to an HPLC system. The column was eluted with a gradient of NaCl as described under "Materials and methods". The eluant was monitored for UV absorbance at 232 nm. The numbered peaks correspond to elution positions of know disaccharide standards: peak 1, ΔHexUA-GalNAc; peak 2, ΔHexUA-GalNAc(6S); peak 3, ΔHexUA-GalNAc(4S); peak 4, ΔHexUA(2S)-GalNAc(6S); peak 5, ΔHexUA-GalNAc(4S, 6S); peak 6, ΔHexUA(2S)-GalNAc(4S); peak 7, ΔHexUA(2S)-GalNAc(4S, 6S).

The disaccharides formed by the action of chondroitinase ABC are shown in Figure 4 and in Table 1. The method used allowed the separation of the following standard disaccharides: ΔUA-GalNAc, ΔUA(2S)-GalNAc, ΔUA-GalNAc(6S), ΔUA-GalNAc(4S), ΔUA(2S)-GalNAc(6S), and ΔUA(2S)-GalNAc(4S) (6S represents 6-O-sulfate). The disaccharide composition of the DSs from the stolidobranchs *H. pallida *and *H. roretzi *are very similar and homogeneous, showing the typical disaccharides detected in the DSs from other species of this order (*S. plicata *and *H. pyriformis*) (Table 1). They consist of about 80% and 20% of the disaccharides ΔUA(2S)-GalNAc(4S) and ΔUA-GalNAc(4S), respectively. Minor amounts of the disaccharides ΔUA-GalNAc, ΔUA(2S)-GalNAc and ΔUA(2S)-GalNAc(6S) are also present (Figure 4 and Table 1).

**Table 1:** Disaccharide composition of the ascidian DSs

	Δ-Disaccharide (Mol%)					
	
Δ-Disaccharide	Order Stolidobranchia				Order Phlebobranchia	
	
	***S.plicata***^**a**^	***H.pallida***^**c**^	***H.pyriformis***^**b**^	***H.roretzi***^**c**^	***P.nigra***^**b**^	***C.intestinalis***^**c**^
ΔHexUA-GalNAc	-	0.25	-	0.7	1.1	5.61
ΔHexUA(2S)- GalNAc	-	-	-	1.2	-	-
ΔHexUA- GalNAc(6S)	1	-	1	-	21.4	25.35
ΔHexUA- GalNAc(4S)	28	18.46	29	20.8	-	30.7
ΔHexUA(2S)- GalNAc(6S)	5	0.54	-	-	76.6	36.1
ΔHexUA(2S)- GalNAc(4S)	66	80.76	70	77.3	-	2.17
ΔHexUA(2S)- GalNAc(4.6S)	-	-	-	-	0.9	-

^a ^Data from reference [6]

^b ^Data from reference [5]

^c ^Data obtained in this work-The areas under the peaks in Fig.4 were integrated to obtain the proportion of the disaccharides.

The DS from the phlebobranch *C. intestinalis *has a more heterogeneous disaccharide composition, when compared with the DSs from stolidobranch species (Figure 4 and Table 1). Whereas disulfated disaccharides prevail in Stolidobranchia DSs, accounting for at least 70% of the polymers, mono-sulfated disaccharides are the major units in the DS from *C. intestinalis*, corresponding to about 55% of the composition. These units are sulfated at either carbon 4 or carbon 6 of the galactosamine, while the mono-sulfated disaccharides in stolidobranch DSs are mainly sulfated at carbon 4 of the galactosamine (Table 1). The main disulfated disaccharide in Phlebobranchia DSs is the ΔUA(2S)-GalNAc(6S), accounting for 36% and 77% in *C. intestinalis *and *P. nigra *DSs, respectively. The DSs from Phlebobranchia ascidians differ in that the 4-sulfated galactosamine units are present in *C. intestinalis *and absent in *P. nigra*.

### Sulfation pattern of ascidians DSs and HCII activity

**Figure 5:**
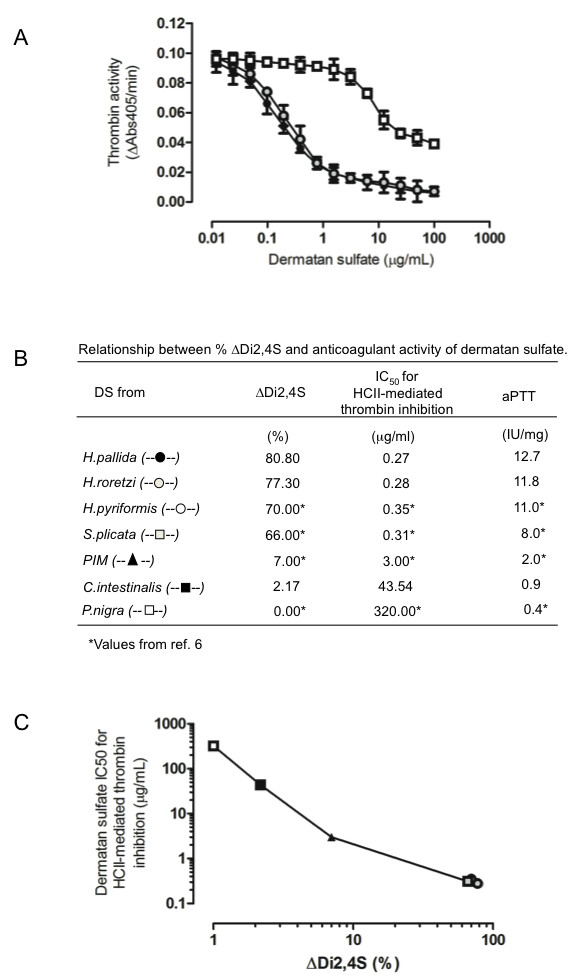
**Direct measurement of the inhibition of thrombin by heparin cofactor II in the presence of the different ascidian DSs**. A, inhibition of thrombin activity by HCII in the presence of DSs from *H. pallida *(- λ -), *H. roretzi *(- λ -), and *C. intestinalis *(- ν -). HCII (68 nM) was incubated with thrombin (15 nM) in the presence of various concentrations of glycans. After 60 seconds, the remaining thrombin or factor Xa activity was determined with a chromogenic substrate (ΔA405/min). The results are expressed as % of thrombin activity. B, IC_50 _(mg/ml) values for thrombin inhibition by HCII in the presence of DSs containing different percentages of 2,4-Disulfated disaccharide units (ΔDi2,4S) obtained from the ascidians *H. pallida *(- λ -), *H. roretzi *(- λ -), *H. pyriformis *(- ○ -), *S. plicata *(- ν -), Porcine intestinal mucosa (PIM) (- σ -), *C. intestinalis *(- ν -) and *P. nigra *(- □ -).

Figure 5 A shows a direct measurement of the inhibition of thrombin by heparin cofactor II in the presence of the different ascidians DSs. The IC_50 _for thrombin inhibition is 0.27, 0.28 and 43.54 μg/ml for the *H. pallida*, *H. roretzi *and *C. intestinalis *DSs, respectively (Figure 5A and 5B). Figure 5B, shows the percentage of 2,4-Di-sulfated units in the DSs from the different ascidian species, their correspondent IC_50 _for thrombin inhibition by HCII, and aPTT values. The HCII activity of the ascidians DS, which is estimated by the IC_50 _(the lower IC_50 _the higher HCII activity), increases with the augment of 2,4-Di-sulfated units (Figure 5B and 5C). Therefore, *H. pallida *DS, which contains ~81% of 2,4-Di-sulfated units has the higher HCII activity, whereas *P. nigra *DS, with 0% of 2,4-Di-sulfated units has the lowest HCII activity. Interestingly, the DSs from Stolidobranchia ascidians have the highest content of 2,4-Di-sulfated units and the higher HCII activity, whereas the opposite is observed for the DSs from Phlebobranchia species.

As shown in Figure 5B, the compositional differences and HCII inhibition activity of most DSs correlates with variations in aPPT values. Thus, higher proportion of 2,4-disulfated units correlates with higher aPTT values.

## Discussion

The subphylum Tunicata comprises the ascidians and two other taxa, Appendicularia and Thaliacea. Tunicates are proposed in some phylogenies as the closest living relatives of the vertebrates [10,11], although most of the recent reconstructions still place the Cephalochordata as the sister group of the back-boned animals [12-14].

In order to study how sulfation of DS evolved in tunicates we compared the disaccharide composition of DS polymers obtained from ascidians of two different orders: Stolidobranchia and Phlebobranchia. The disaccharide analysis indicated a high content of disulfated disaccharide units in the DSs from both taxa. Interestingly, the degree of DS sulfation decreased on Phlebobranchia clade. Thus, 76% of the disaccharide units in the DSs from stolidobranch ascidians are disulfated, compared to 53% in the DSs from the phlebobranch. In fact, the two taxa probably diverged early during the evolution of tunicates. Phlebobranch ascidians have a simpler pharynx, without the folds present in the stolidobranch. Additionally, the phlebobranch blood contains usually vanadium, while stolidobranch has iron instead [16]. Other important distinctions are present in terms of the development of the atrial cavity and position of the gonads [15]. Also, the aforementioned recent hypotheses about the evolution of the tunicates place the thaliaceans within the Phlebobranchia. Therefore, the study of the DS composition of thaliaceans will be relevant to understand the evolutionary history of the group.

The sulfation pattern of the tunicate DS disaccharides showed interesting results, pointing to an evident structural difference: DS from phlebobranch ascidians contain mainly 2,6-sulfated disaccharides, whereas DSs from stolidobranch ascidians contain mostly 2,4-sulfated disaccharides. Although information about sulfation pattern in DS from other Deuterostomia is scanty, both types of sulfation are found in vertebrates. While 2,4-sulfated disaccharides are commonly found in extracellular DS proteoglycans, such as decorin and biglycan present in different tissues as tendon, cartilage or skin [17], significant yet small proportions (5%) of 2,6-sulfated units have been found in mouse or pig brain [18,19] and the biosynthetic regulation of the expression of 2,6-sulfated disaccharides by 6-O-sulfotransferases, 2-O-sulfotransferase and C5-epimerase might play critical roles in the development of the hippocampus and cerebellum [4]. Considering those data, our results point to the 2,4-sulfation as a conserved feature along Chordata and the predominance of 2,6-sulfated disaccharides as the derived condition. Further investigation on the DS structure of echinoderms and hemichordates should clarify this issue.

In vertebrate chordates, four groups of sulfotransferases are involved in the modification of CS/DS: (1) DS-specific 4-O-sulfotransferase; (2) chondroitin 6-O-sulfotransferase; (3) uronyl 2-O-sulfotransferase; (4) GalNAc-4-sulfate 6-O-sulfotransferase. Interestingly, a comprehensive survey of sulfotransferases in the ascidian *C. intestinalis *showed similarity to vertebrate CS/DS-specific sulfotransferases. From eight *C.intestinalis *proteins found to be closely related to DS/CS 4-O-sulfotransferases, four were proposed to have arisen by duplication during the evolution of tunicates. Although eight genes encoding C6ST-like proteins were also found in *C. intestinalis *genome, their evolutionary history was not clear. It was suggested that divergence of the C6ST family genes has occurred independently during the evolution of tunicate, cephalochordate and vertebrate lineages [20]. *C. intestinalis *genome presented also four genes encoding urosyl 2-O-sulfotransferases, one of them very closely related to human 2-O-sulfotransferases. However, a GalNAc-4-sulfate 6-O-sulfotransferase homologous gene was not found in *C. intestinalis *genome. In fact, no 4,6-sulfated disaccharides were found in DSs from *C. intestinalis *or other ascidians analyzed in the present work, despite the high content of 4-O-sulfated N-acetylgalactosamine in stolidobranch ascidians and the presence of these units also in *C. intestinalis*. On the other hand, albeit up 90% of disaccharide units in DS from *P. nigra *contains 6-O-sulfated N-acetylgalactosamine, no 4-O-sulfated units were found.

The differences in the sulfation patterns from *C. intestinalis *and *P. nigra *may also result from divergent evolution, as these species are not closely related within the Phlebobranchia. Our results on the structure of DSs from ascidians add to Tetsukawa's results, suggesting different mechanisms for GAG sulfation according to taxa, which may have been emerged via gene duplication, occurring independently in each group and leading to different types of 4-O-sulfotransferases and 6-O-sulfotransferases in ascidians and vertebrates, with distinct substrate specificities.

The high affinity binding of DS to HCII requires a rare sequence consisting of [IdoA(2S)-GalNA(4S)]_3 _[3]. Interestingly, DS with higher sulfation levels bind poorly to HCII, suggesting significant specificity of interaction [6,21]. Corroborating with this idea, recent work has shown that a specific geometry of the [IdoA(2S)-GalNA(4S)]-hexasaccharide, which forms a 2_1_-helix topology (2_1_-°S_2_.^1^C_4_.^1^C_4_), is required for high affinity binding to HCII [22]. This geometry allows the interaction of the [IdoA(2S)-GalNA(4S)]-hexasaccharide with key aminoacids involved in the binding to HCII and the correct orientation for the binding to exocite II of thrombin. Extention of the high-affinity sequence in DS polymers is expected to increase the binding and the activity of HCII [22].

In the present, work we observed that ascidian DSs with increasing content of 2,4-sulfated disaccharides showed increasing HCII activity, (Figure 5A-B). Therefore, in consonance with the previous paragraphy, it is possible to suggest that in the HCII-active DSs, [IdoA(2S)-GalNA(4S)]-disaccharides are clustered in 2_1_-helix topology (2_1_-°S_2_.^1^C_4_.^1^C_4_) sequences with inceasing lengh.

Finally, an interesting characteristic of ascidians is their ability to rapid vessel regeneration [23]. Such event, taken together with the fact that in vertebrates DS have been demonstrated to be involved in wound repair, raises intriguing questions concerning the molecular mechanisms underlying angiogenesis. Is the presence of oversulfated DS in ascidians related to fast vessel regeneration by binding to growth factors?

Furthermore, the drastic difference between the DS disaccharide units from phlebobranch and stolidobranch ascidians may also be related to an angiogenesis event. The alteration between a 2,4 disaccharide from Stolidobranchia to a 2,6 disaccharide in Phlebobranchia might be involved in the branchial vessel organization. One of the main differences between these two ascidian orders is the pharyngeal structure. Phlebobranch ascidians possess a simpler pharynx, without folds and usually with less internal vessels. On the other hand, stolidobranch ascidians have a more complex structure, with a folded pharynx and a larger number of internal vessels. The DS structural differences from both ascidian orders could be related to the pharynx vascular organization.

Notably the evolution of glycosaminoglycans in animals including invertebrates as well as vertebrates has also recently been reviewed in terms of their structure and biological activities [24].

## Conclusions

Our results indicate that in ascidians the position of sulfation on the GalNAc in the disaccharide [→4IdoA(2S)β-1→3GalNAcβ-1→] is directly related to the taxon and that the 6-O sulfation is a novelty apparently restricted to the Phlebobranchia. We also show that an increase in the content of [→4IdoA(2S)β-1→3GalNAc(4S)β-1→] disaccharide units in DS accounts for an increased HCII-stimulating activity.

## Methods

### Isolation of ascidian DSs

The ascidians *H. pallida *and *P. nigra *were collected in Angra dos Reis, Rio de Janeiro, Brazil; *S. plicata *was collected at Praia da Urca, Rio de Janeiro, Brazil; *C. intestinalis *was obtained from the Marine Biological Laboratory, Woods Hole, Massacchussets, USA; *H. roretzi *was obtained from the Japanese company (Maruha Nichiro Holdings, Inc., Tokyo). To extract the glycans, the animals had the tunic removed and were maintained in acetone at room temperature overnight. Tissues were dried at 60°C, homogenized, suspended in twenty-volumes of digestion buffer (50 mM sodium acetate, 5 mM EDTA, 10 mM cysteine, pH 5.0), containing 10% papain (w/w), and incubated at 60°C, overnight. Soluble glycans were precipitated with two-volumes of ethanol and fractionated in an anion exchange chromatography on a Mono-Q-FPLC column (HR 5/5) (Amersham Pharmacia Biotech), equilibrated in 20 mM Tris/HCl (pH 8.8), containing 4 mM EDTA. The column was developed with a linear gradient of 0-2.0 M NaCl in the same buffer, at a flow rate of 1 mL/min. The fractions from the column were monitored by metachromatic property with dimethylmethylene blue, at 525 nm [25]. The metachromatic fractions were pooled, dialyzed, lyophilized and analyzed by agarose gel electrophoresis.

### Agarose gel electrophoresis

The ascidian glycans were analyzed by agarose gel electrophoresis, as described [5]. Briefly, GAGs (~15 mg) were applied to a 0.5% agarose gel and ran for 1 h at 100 V in 0.05 M 1,3-diaminopropane/acetate (pH 9.0). GAGs were fixed in the gel with 0.1% *N*-cetyl-N,N,N-trimethylammonium bromide solution. After 12 h the gel was dried and stained with 0.1% toluidine blue in acetic acid/ethanol/water (0.1:5:5, v/v).

Polyacrylamide gel electrophoresis-The molecular weight of the ascidian DSs was estimated by polyacrylamide gel electrophoresis. DS samples (~10 mg) were applied to a 7.5% 1-mm-thick polyacrylamide gel slab in 0.02 M sodium barbital (pH 8.6). After electrophoresis (100 V for 30 min), the gel was stained with 0.1% toluidine blue in 1% acetic acid and then washed for about 4 h in 1% acetic acid. The molecular weight markers were the same as those used previously [26].

### Enzymatic treatments with chondroitin lyases

The glycans extracted from ascidians (~20 mg) were incubated with 0.005 U of chondroitin AC or ABC lyases, as described previously [6]. Intact and enzyme-degraded glycans were analyzed by agarose gel electrophoresis, as described earlier.

Deaminative cleavage with nitrous acid - deaminative cleavage was performed by nitrous acid at pH 1.5, as described [27]. Briefly, total glycans extracted from ascidians tissues (~20 μg) were incubated with 5 μL of fresh generated HNO_2 _at room temperature for 90 min. The reaction mixtures were then neutralized with 1.0 M Na_2_CO_3_. Intact and nitrous acid-degraded glycans were analyzed by agarose gel electrophoresis.

### Analysis of the disaccharides formed by digestion of the dermatan sulfate with chondroitin ABC lyase

The purified DSs from the ascidians were exhaustively degraded with chondroitin ABC lyase, as described earlier. The disaccharides formed were recovered by Superdex 75 (Amersham Pharmacia Biotech), which was connected to a HPLC system from Shimadzu (Tokyo, Japan). The column was equilibrated with an aqueous solution of 20% acetonitrile (pH 3.5) and developed at a flow rate of 0.25 mL/min. Fractions of 0.5 mL were collected and checked for absorbance at 232 nm. Fractions containing disaccharides were pooled, freeze-dried and analyzed by strong anion-exchange chromatography on a 25-cm Spherisorb-SAX column (Sigma-Aldrich), linked to a HPLC system. The column was developed with a linear gradient of 0 - 1.0 M aqueous NaCl (pH 3.5) at a flow rate of 0.5 ml/min. The elution of the disaccharides was followed by absorbance at 232 nm, and identified by comparison with the elution positions of standard disaccharides.

### Hexuronic acid determination

The content of DS was estimated measuring hexuronic acid in the purified samples by the carbazole reaction [28].

### Activated partial thromboplastin time (aPTT) assay

aPTT clotting assays were carried out as described previously [6]. Briefly, normal human plasma (90 μL) was incubated with 10 μL of DS at several dilutions and 100 μL of cephalin. After three minutes at 37°C, 100 μL of 0.25 M CaCl_2 _were added to the mixtures and the clotting time was recorded. The activity was expressed as units/mg using a standard unfractionated heparin (180 U/mg) curve.

### Heparin cofactor II-mediated inhibition of thrombin by ascidian DSs

The inhibitory activity of ascidian DSs was measured in 96-well plate. Briefly, 30 μL of 68 nM HCII in 0.02 M Tris-HCl, pH 7.4/0.15 M NaCl/PEG 1 mg/mL were incubated with 30 μL of ascidian DSs at different concentrations. After incubating for 2 minutes, 10 μL of 50 μM thrombin was added. Thrombin activity was measured using a synthetic chromogenic substrate (S-2238 from DiaPharma Group). The HCII-mediated thrombin inhibition in presence of DSs was expressed as the percentage of thrombin activity during 9 minutes. All DSs were assayed twice in triplicate and expressed as the mean ± the standard deviation.

## Abbreviations

-UA(2S): DHexUA, -4,5-unsaturated hexuronic acid 2-sulfate; -UA: -4,5-unsaturated hexuronic acid; GalNAc(4S): GalNAc(6S) and GalNAc(4,6-diS), derivatives of 2-acetamido-2-deoxy-galactose bearing a sulfate ester at position 4, at position 6, and at both positions, respectively; -L-IdoA: -L-iduronic acid; -L-IdoA(2S): -L-iduronic acid 2-sulfate; FPLC: fast protein liquid chromatography; HPLC: high performance liquid chromatography; aPTT: activated partial thromboplastin time.

## Authors' contributions

EOK, PL and CPV carried out the extraction, isolation and characterization of the ascidians glycosaminoglycans. EOK and PL also carried out the HCII activation assay and drafted the manuscript. XB and KS participated in the extraction, isolation and characterization of the *H. roretzi *glycosaminoglycans and in the critical reading of the manuscript. TL participated in the phylogenetic analysis of the ascidians and in the critical reading of the manuscript. MSGP conceived of the study, and participated in its design and coordination and helped to draft the manuscript. All authors read and approved the final manuscript.
