# Correction: Dermatan sulfate in tunicate phylogeny: Order-specific sulfation pattern and the effect of [→4IdoA(2-Sulfate)β-1→3GalNAc(4-Sulfate)β-1→] motifs in dermatan sulfate on heparin cofactor II activity

**DOI:** 10.1186/1471-2091-12-37

**Published:** 2011-07-13

**Authors:** Eliene O Kozlowski, Paula C Lima, Cristina P Vicente, Tito Lotufo, Xingfeng Bao, Kazuyuki Sugahara, Mauro SG Pavão

**Affiliations:** 1 Laboratório de Bioquímica e Biologia Celular de Glicoconjugados, Hospital Universitário Clementino Fraga Filho and Programa de Glicobiologia, Instituto de Bioquímica Médica, Universidade Federal do Rio de Janeiro, Rio de Janeiro, RJ 21941-913, Brasil; 2 Department of Anatomy, Cellular Biology, Physiology and Biophysics, Instituto de Biologia, Universidade Estadual de Campinas, São Paulo, Brasil; 3 Laboratório de Ecologia Animal, Instituto de Ciências do Mar, Universidade Federal do Ceará, Av. Abolição 3207, Fortaleza, CE, 60165-081, Brasil; 4 Department of Biochemistry, Kobe Pharmaceutical University, Kobe 658-8558, Japan; 5 Glycobiology Unit, Tumor Microenvironment Program, Cancer Research Center, Sanford-Burnham Institute for Medical Research, 10901 North Torrey Pines Road, La Jolla, California 92037, USA; 6 Laboratory of Proteoglycan Signaling and Therapeutics, Hokkaido University Graduate School of Life Science, Frontier Research Center for Post-Genomic Science and Technology Graduate, Sapporo 001-0021, Japan

## Abstract

After the publication of the work entitled "Dermatan sulfate in tunicate phylogeny: Order-specific sulfation pattern and the effect of [→4IdoA(2-Sulfate)β-1→3GalNAc(4-Sulfate)β-1→] motifs in dermatan sulfate on heparin cofactor II activity", by Kozlowski et al., *BMC Biochemistry *2011, **12:**29, we found that the legends to Figures 2 to 5 contain serious mistakes that compromise the comprehension of the work. This correction article contains the correct text of the legends to Figures 2 to 5.

## Correction

After the publication of the work entitled "Dermatan sulfate in tunicate phylogeny: Order-specific sulfation pattern and the effect of [→4IdoA(2-Sulfate)β-1→3GalNAc(4-Sulfate)β-1→] motifs in dermatan sulfate on heparin cofactor II activity", by Kozlowski et al., *BMC Biochemistry *2011, **12:**29 [1], we found that the legends to Figures 2 to 5 contain serious mistakes that compromise the comprehension of the work. We would like to correct the legends to these figures as follows:

Figure two: **Purification of the dermatan sulfate (DS) from *H. pallida *(A and C) and *C. intestinalis *(B and D) on a Mono QFPLC column**. A and B, the total polysaccharides extracted from ascidians were applied to a Mono Q-FPLC column and purified as described under "Materials and methods". Fractions were assayed by metachromasia (open circle), and NaCl concentration (- -). The fractions under the peaks indicated by horizontal bracket were pooled, denominated P1.2 and P1.6 (A) or P0.9, P1.2 and P1.6 (B), dialyzed against distilled water and lyophilized. C and D, ~15 mg of each peak from Mono Q-FPLC column were applied to a 0.5% agarose gel and run for 1 h at 100 V in 0.05 M 1,3-diaminopropane/acetate (pH 9.0). The polysaccharides in the gel were fixed with 0.1% N-cetyl-N,N,N-trimethylammonium bromide solution. After 12 h, the gel was dried and stained with 0.1% toluidine blue in acetic acid/ethanol/water (0.1:5:5, v/v).

Figure three: **Agarose and polyacrylamide gel electrophoresis of ascidians and mammalian dermatan sulfate**. A, purified dermatan sulfate (~15 mg) from *S. plicata, H. pallida, H. rotetzi, P. nigra *and *C. intestinalis *were applied to a 0.5% agarose gel and run for 1 h at 100 V in 0.05 M 1,3-diaminopropane/acetate (pH 9.0). Glycosaminoglycans were fixed with 0.1% N-cetyl-N,N,Ntrimethylammonium bromide solution. After 12 h, the gel was dried and stained with 0.1% toluidine blue in acetic acid/ethanol/water (0.1:5:5, v/v). To a standard, a mixture of mammalian glycosaminoglycans containing 10 mg each of chondroitin 4-sulfate (CS), dermatan sulfate (DS) and heparin (Hep) were applied in agarose gel. B, purified dermatan sulfate (~15 mg) from *S. plicata, H. pallida, H. rotetzi, P. nigra *and *C. intestinalis *were applied to 6% 1-mm-thick polyacrylamide gel slab in 0.02 M sodium barbital (pH 8.6) and run for 30 min at 100 V. After electrophoresis the gel was stained with 0.1% toluidine blue in 1% acetic acid and then washed for about 4 h in 1% acetic acid. The molecular mass (M.W.) markers were high molecular mass dextran sulfate (S1, ~500 kDa), chondroitin 6-sulfate (S2, ~54 kDa), chondroitin 4-sulfate (S3, ~36 kDa) and low molecular mass dextran sulfate (S4, ~8 kDa). Stolidobranchia and Phlebobranchia are the taxonomic Orders of the ascidians.

Figure four: **Strong anion-exchange HPLC analysis of the disaccharides formed by chondroitin ABC lyase digestion of ascidians dermatan sulfate**. The disaccharides formed by exhaustive action of chondroitin ABC lyase on dermatan sulfate from *H. pallida, H. roretzi *and *C. intestinalis *were applied to a 25-cm × 4.6-mm Spherisorb-SAX column, linked to an HPLC system. The column was eluted with a gradient of NaCl as described under "Materials and methods". The eluant was monitored for UV absorbance at 232 nm. The disaccharides of the ascidians DS were identified by comparing the elution positions with those of standard disaccharides: deltaDi-0S, deltaHexUA-GalNAc; deltaDi-6S, deltaHexUA-GalNAc(6S); deltaDi-4S, deltaHexUA-GalNAc(4S); deltaDi-2,6S, deltaHexUA(2S)-GalNAc(6S); deltaDi-2,4S, deltaHexUA(2S)-GalNAc(4S).

Figure five: **Direct measurement of the inhibition of thrombin by heparin cofactor II in the presence of the different ascidian DSs**. A, inhibition of thrombin activity by HCII in the presence of DSs from *H. pallida *(- closed circle/black diamonds -), *H. roretzi *(- grey circle -), and *C. intestinalis *(- open square -). HCII (68 nM) was incubated with thrombin (15 nM) in the presence of various concentrations of glycans. After 60 seconds, the remaining thrombin or factor Xa activity was determined with a chromogenic substrate (deltaA405/min). B, Table showing the values of the percentage of deltaDi2,4S, the IC_50 _(mg/ml) for HCII-mediated thrombin inhibition and the aPTT of the ascidians DSs. C. Relationship between IC_50 _(mg/ml) values for HCII-mediated thrombin inhibition in the presence of DSs containing different percentages of deltaDi2,4S units obtained from the ascidians *H. pallida *(- closed circle -), *H. roretzi *(- grey circle -), *H. pyriformis *(- open circle -), *S. plicata *(- grey squate -), Porcine intestinal mucosa (PIM) (- close triangle -), *C. intestinalis *(- closed square -) and *P. nigra *(- open square -). 

We apologise for any inconvenience that this inaccuracy in the figure legends in the article [1] might have caused.
